# Metagenomic analysis examines oral microbiome changes and interplay with immune response following prenatal total oral rehabilitation

**DOI:** 10.1186/s12967-023-03997-9

**Published:** 2023-03-04

**Authors:** Tong Tong Wu, Michael Sohn, Samantha Manning, Robert Beblavy, Steven Gill, Sally Quataert, Shruti Vasani, Hoonji Jang, Yan Zeng, Jennifer Bruno, Adriana Vazquez, Kevin Fiscella, Jin Xiao

**Affiliations:** 1 Department of Biostatistics and Computational Biology, University of Rochester Medical Center, Rochester, NY USA; 2 Immunology and Microbiology, University of Rochester Medical Center, Rochester, NY USA; 3 Eastman Institute for Oral Health, University of Rochester Medical Center, Rochester, NY USA; 4 Temple University Maurice H. Kornberg School of Dentistry, Philadelphia, USA; 5 University of Puerto Rico-Arecibo Campus, Arecibo, PR USA; 6 Department of Family Medicine, University of Rochester Medical Center, Rochester, NY USA

**Keywords:** Pregnancy, Prenatal oral health care, Bacteriome, Mycobiome, Metagenomics, Immune markers

## Abstract

**Background:**

Suboptimal maternal oral health during pregnancy is potentially associated with adverse birth outcomes and increased dental caries risks in children. This study aimed to assess the oral microbiome and immune response following an innovative clinical regimen, Prenatal Total Oral Rehabilitation (PTOR), that fully restores women’s oral health to a “disease-free status” before delivery.

**Methods:**

This prospective cohort study assessed 15 pregnant women at baseline and 3 follow-up visits (1 week, 2 weeks, and 2 months) after receiving PTOR. The salivary and supragingival plaque microbiomes were analyzed using metagenomic sequencing. Multiplexed Luminex cytokine assays were performed to examine immune response following PTOR. The association between salivary immune markers and oral microbiome was further examined.

**Results:**

PTOR was associated with a reduction of periodontal pathogens in plaque, for instance, a lower relative abundance of *Tannerella forsythia* and *Treponema denticola* at 2 weeks compared to the baseline (p < 0.05). The alpha diversity of plaque microbial community was significantly reduced at the 1-week follow-up (p < 0.05). Furthermore, we observed significant changes in the *Actinomyces defective*-associated carbohydrate degradation pathway and *Streptococcus Gordonii*-associated fatty acid biosynthesis pathway. Two immune markers related to adverse birth outcomes significantly differed between baseline and follow-up. ITAC, negatively correlated with preeclampsia severity, significantly increased at 1-week follow-up; MCP-1, positively correlated with gestational age, was elevated at 1-week follow-up. Association modeling between immune markers and microbiome further revealed specific oral microorganisms that are potentially correlated with the host immune response.

**Conclusions:**

PTOR is associated with alteration of the oral microbiome and immune response among a cohort of underserved US pregnant women. Future randomized clinical trials are warranted to comprehensively assess the impact of PTOR on maternal oral flora, birth outcomes, and their offspring’s oral health.

**Supplementary Information:**

The online version contains supplementary material available at 10.1186/s12967-023-03997-9.

## Introduction

In mothers, suboptimal and untreated prenatal oral health care is an essential contributor to poor maternal oral health, potential adverse birth outcomes, and increased risk for early childhood caries in children [1–3]. First, pregnancy increases the risk of oral diseases in the mother (periodontal disease and dental caries) due to physiological, hormonal, and dietary changes [4], which often leads to mothers needing more routine/urgent oral health services than when they are not pregnant. Second, there are well-documented observational associations between poor maternal oral health and increased risk for preterm/low-birth-weight deliveries [5, 6]. Third, poor maternal oral health is associated with an increased risk for dental caries in children [7]. Mother’s oral health impacts children’s oral health at multiple levels. In the Fisher-Owens conceptual model for child oral health, the mother heavily influences the child from biological and environmental aspects [8]. This association likely results from a substantial influence of maternal oral health/behaviors on children’s oral health and well-documented maternal-infant transmission of oral microorganisms [9].

Dental caries is an infectious disease caused by oral pathogens, with diet and oral hygiene as additional contributors. When assessing the longitudinal influence of the mothers on the establishment of children’s oral microbiome, the impact first occurs when the babies are still in the womb [10]. Several oral microorganisms, such as *Streptococcus*, *Fusobacterium*, *Neisseria*, *Prevotella*, and *Porphyromonas*, were found in the human placenta [11, 12]. In addition, studies have detected common oral bacteria in the amniotic fluid [11], and the placental microbiome resembles the pregnant women’s oral microbiome more than the gut microbiome [13]. After the child is born, the mother’s influence is also shown through some key oral pathogens’ transmission. For example, 70% of mothers and children share genetically identical *S. mutans* strains [14]. For *C. albicans*, one of the early colonizers, our study found that > 60% of mothers and preschool children share genetically identical strains [15].

Poor maternal and child oral health has become an increasingly recognized public health problem in the US (and worldwide), affecting the most vulnerable population who are racially and socioeconomically deprivileged [16–18]. With the intent to improve maternal and children’s oral health, we proposed a new therapy regimen—Prenatal Total Oral Rehabilitation (PTOR), that targets the critical prenatal period to fully restore women’s oral health to a “disease-free status” before the delivery. The PTOR includes a comprehensive examination, dental cleaning, fillings to restore dental caries, tooth extractions, root canal therapy, and periodontal treatment as needed. Our previous study among 15 pregnant women and their controls revealed that PTOR was associated with improved oral health conditions, perinatal oral health literacy, and a reduction in S. mutans carriage within a 2-month follow-up period [3]. Here, we comprehensively assessed the impact of PTOR on the maternal oral flora other than *S. mutans* and *Candida*, and the immune response among pregnant women.

## Materials and methods

### Study population

This study included 15 pregnant women who received PTOR at the Perinatal Dental Clinic at the University of Rochester Medical Center (URMC) Eastman Institute for Oral Health (EIOH). The PTOR treatment was completed in 1–2 clinical visits. The reported study was approved by the University of Rochester Research Subject Review Board (#4628). All participants were informed of the study objects and protocols and gave written consent prior to study activities.

### Eligibility

Individuals who met the following criteria were enrolled.

The inclusion criteria were: (1) ≥ 18 years old; (2) pregnant, ≤ 28 gestational weeks; (3) 1–5 untreated carious teeth. (4) did not receive dental cleaning in the past 5 months. (5) < 4 mm periodontal pocket depth for all teeth.

The exclusion criteria were: (1) decisional impairment; (2) received oral and/or systemic antifungal therapy within 90 days of the baseline study visit; (3) required premedication before dental treatment; (4) > 8 missing teeth, except third molars and orthodontically extracted teeth; (5) removable dental prosthesis; (6) orofacial deformity (e.g., cleft lip/palate); (7) had severe systemic diseases (e.g., HIV).

### Data collection, examination and sample collection

The participants were examined at four time points: (1) baseline visit (V1), before receiving PTOR; (2) 1 week after receiving PTOR (V2); (3) two weeks (V3) and two months (V4) after PTOR. Demographic-socioeconomic characteristics and oral hygiene behavior were collected using a questionnaire, detailed previously [3]. In addition, medical background, medications, and smoking status were self-reported and confirmed by electronic medical records.

A comprehensive oral examination was performed at each visit by one of two calibrated dentists in a dedicated examination room at the URMC, using standard dental examination equipment, materials, and supplies. Caries were scored using DMFT (decayed, missing, and filled teeth) and the International Caries Detection and Assessment System (ICDAS) [19]. Bleeding on probing (BOP) was used to assess the gingival inflammation. Supragingival plaque was assessed using the Plaque Index (PI) described by Löe [20]. Inter- and intra-examiner agreement for the evaluated criteria was calculated by Kappa statistics and exceeded 90% at the calibration.

Saliva/plaque sample collection was detailed previously [3]. The study participants spit approximately 2 ml of whole non-stimulated saliva into a sterile 50 ml centrifuge tube. Study subjects were instructed not to eat, drink or brush their teeth 2 h before oral sample collection prior to their study visit. Supragingival plaques from the whole dentition were collected using a sterilized periodontal scaler. The plaque samples were resuspended in 1 ml of a 0.9% sodium chloride solution in a sterilized Eppendorf tube. All samples were transported to the lab within 2 h of collection and stored in a – 80 ^°^C freezer.

### DNA extraction

The whole genome shotgun DNA Extraction Samples were extracted with Dneasy PowerSoil Pro Kit (Qiagen, Maryland, US), automated for high throughput on the QiaCube HT instrument (Qiagen, Maryland, US), using Powerbead Pro Plates (Qiagen, Maryland, US) with 0.5 mm and 0.1 mm ceramic beads. Samples were quantified with Quant-iT PicoGreen dsDNA Assay Kits (Invitrogen^™^, ThermoFisher, US).

### Library preparation and metagenomic sequencing

Libraries were prepared with a procedure adapted from the Nextera Library Prep kit (Illumina, San Diego, California). For Deep Sequencing, libraries were sequenced on an Illumina NovaSeq using paired-end 2 × 150 reads (Illumina, San Diego, California). DNA sequences were filtered for low quality (Q-Score < 30) and length (< 50), and adapter sequences were trimmed using cutadapt. Fastq files were converted to a single fasta using shi7. Sequences were trimmed to a maximum length of 100 bp prior to alignment. The library size of the sequenced files is shown in Additional file 1: Figure S1. The Median Quality Scores are shown in Additional file 1: Figure S2.

### OTU and functional genome content (DeepSeq)

DNA sequences were taxonomically classified using the MetaPhlAn2 analysis tool MetaPhlAn2 maps reads to clade-specific marker genes identified from ~ 17,000 reference genomes and estimates clade abundance within a sample from these mappings. The rarefaction curve is shown in Additional file 1: Figure S3. Functional profiling was carried out using the HUMAnN2 analysis pipeline. HUMAnN2 uses a combination of pangenome mapping, results from MetaPhlAn2, and translated BLAST searches using Diamond and the UniRef90 database to estimate the abundance of genes in a sample, as well as the abundances of the complete functional pathways.

### Salivary cytokine level

Cytokine/chemokine assessment for 25 analytes was performed in the University of Rochester Human Immunology Center Core Lab facility on saliva samples collected at 4 time points (Baseline, 1-week, 2-week and 2-month after PTOR). Samples were centrifuged for 5 min at 125000 rcf at 4 °C prior to incubation of the sample at neat concentration overnight at 4 °C in two multiplexed magnetic bead array assays, an 18-Plex Milliplex MAP high sensitivity human cytokine panel (Cat#HSTCMAG-28SK) for GM-CSF, IFNg, IL-1B, IL-2, IL-4, IL-5, IL-6, IL-7, IL-8, IL-10, IL-12(p70), IL-13, IL-17A, IL-23, ITAC, MIP-1a, MIP-1B, and TNFα and a 7-Pex Milliplex MAP human cytokine/chemokine panel (Cat#HCYTOMAG-60K) for Eotaxin, IL-1a, IL-1RA, IL-15, IP-10, MDC, and MCP-1. Both assays were performed following kit instructions and read on a Luminex 200 instrument. Results were reported in pg/mL based on standard curve values.

### Statistical analysis

For alpha diversity, the Shannon index was used to capture the richness (number of types of organisms) and evenness (uniformity across organisms) for each participant at each time point. Alpha diversity across time was analyzed using a mixed-effects model for repeated measures (MMRM) with an unstructured covariance matrix [21]. For beta diversity, the Bray-Curtis distance was used, and the results were visualized using principal coordinate analysis (PCoA) and assessed using PERMANOVA with permutations blocked in participants [22]. To determine differentially abundant (DA) pathways and the corresponding DA taxa, an MMRM with an unstructured covariance matrix was used for each pathway, and then for an identified DA pathway, an MMRM with an unstructured covariance matrix was used for each taxon to determine DA taxa. All analyses included race, Decayed teeth (DT), frequency of brushing, and BOP as covariates. For multiple testing correction, the Benjamini-Hochberg procedure was used to control false discovery rate (FDR) at a 5% level [23].

To assess the PTOR-related longitudinal association between immune markers and possibly related factors, we first fitted linear mixed effects models with random intercepts for participants of salivary cytokine levels (pg/ml) of 10 immune markers that had valid detection value over time on demographic and clinic factors. These immune markers were Eotaxin, MDC, IL15, IL1RA, IL1a, IP10, MCP1, ITAC, IL1B, and IL8. The model allows us to compare the cytokine levels of immune markers at follow-up visits to those at baseline by setting the time points as categorical. Other variables included were race, education, vaginal yeast infection, work status, whether brushing teeth twice daily, gestational age (equal or over 20 weeks), ICDAS level, BOP, pain score, and the number of decayed teeth. The 10 models were fitted using statistical software SAS 9.4 (SAS Institute Inc, Cary, NC). We then assessed the association between the species and the immune markers using Lasso-penalized linear mixed effects models with random intercepts using saliva and plaque samples, respectively. The microbiome data in both samples, together with the same set of covariates as in the previous models, were used as candidate variables. The tuning parameters were chosen using Jones’ BIC criterion [24] for longitudinal data. A p-value less than 0.05 (p < 0.05) was considered statistically significant.

## Results

The demographic-socioeconomic-medical-oral background information of 15 pregnant women was reported previously [3]. Briefly, 73% of the pregnant women were African American, and 27% had college or more education. The average decayed teeth number was 2.4 (SD 0.9) before receiving PTOR. The majority of the pregnant women were healthy, with a few individuals with hypertension (13%), diabetes (7%), and smoking during pregnancy (7%). The gestational age of the pregnant women when they entered the study was 26.1 ± 7.5 weeks.

### Oral microbial diversity changes following PTOR

**Fig. 1:**
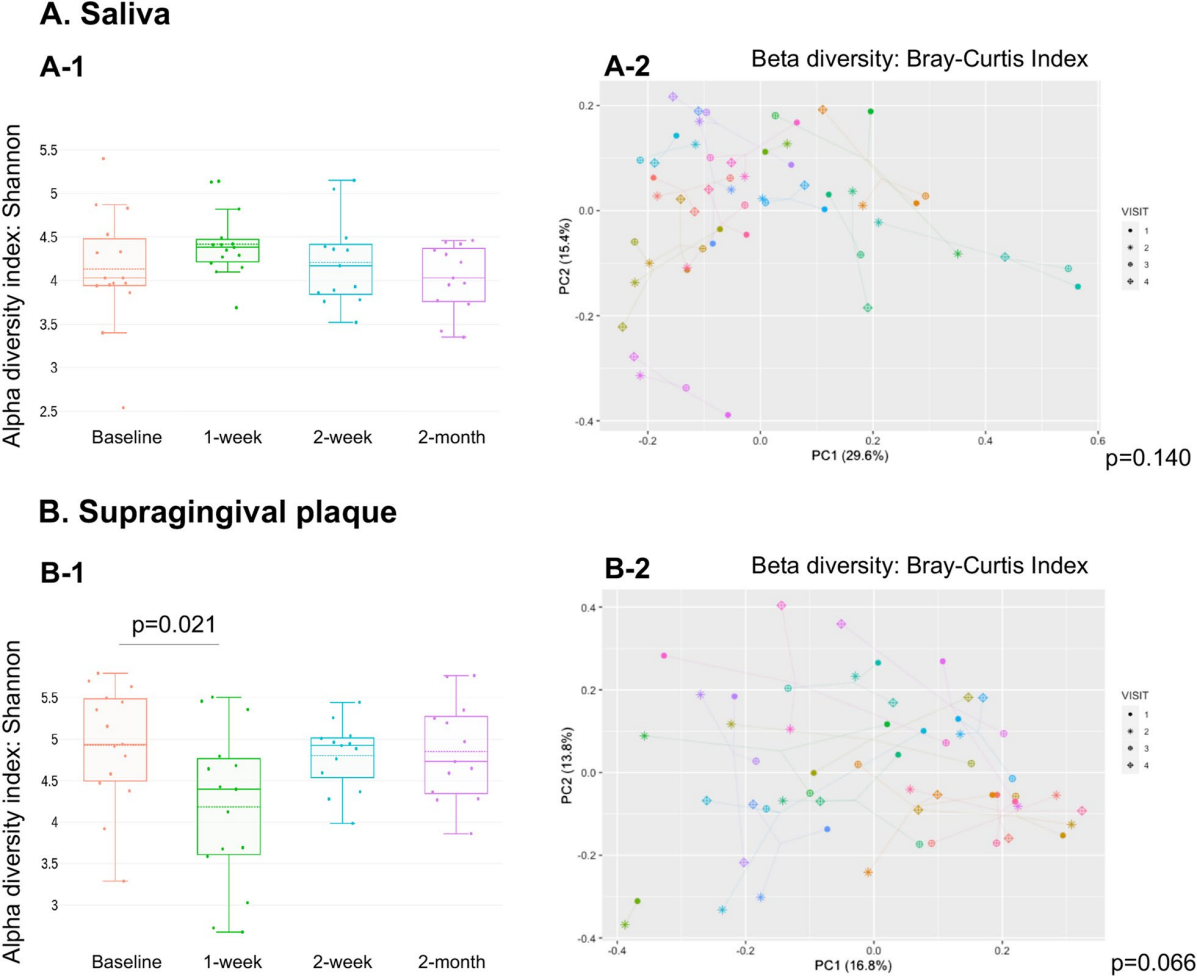
Changes of oral microbiome diversity following Prenatal Total Oral Rehabilitation (PTOR). Microbial variation between PTOR visits measured by Shannon (A1, B1). MMRM was used for statistical analysis. Principle coordinate analysis (PCOA) plot was generated using OTU metrics based on beta diversity Bray–Curtis index (A1, B2). PERMANOVA was used for statistical analysis

**Fig. 2:**
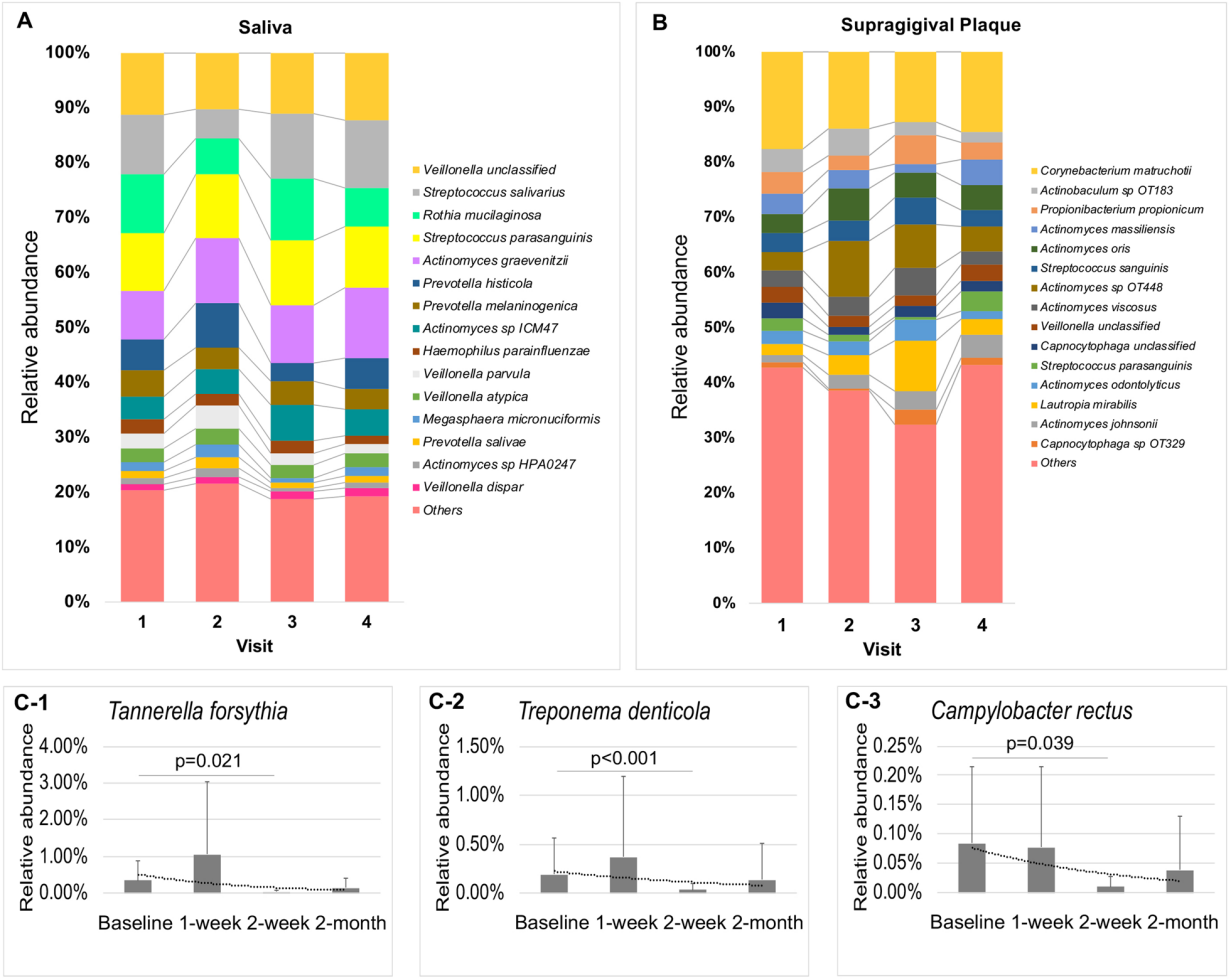
Oral microbiome relative abundance following Prenatal Total Oral Rehabilitation (PTOR). **A** Top 15 species-level microbial relative abundance in saliva. **B** Top 15 species-level microbial relative abundance in supragingival plaque. **C** Relative abundance of periodontal pathogens in supragingival plaque

PTOR did not alter the alpha diversity of the salivary microbiome (Fig. 1A-1); however, it was associated with a less diverse plaque microbial community after PTOR (V2 vs. V1, p = 0.021) (Fig. 1B-1). The reduction of alpha diversity in the plaque microbiome was short-term (within one week); the effect became insignificant two weeks and two months post-PTOR. Differences in saliva and plaque beta diversity were observed in Fig. 1A-2 and B-2. Interestingly, although without statistical significance, the plot indicates that the V2 microbiome in saliva and plaque was far apart from the V1 microbiome, and V3/V4 tends to bound back closer to V1 (saliva p = 0.140, plaque p = 0.066).

### Relative abundance changes following PTOR

A shift of microbial profiling occurred following receiving PTOR (Fig. 2A top 15 species in saliva and Fig. 2B top 15 species in plaque). The relative abundance of cariogenic pathogen *Streptococcus parasanguinis* in supragingival plaque was significantly reduced in the short term (within one-week post-PTOR) (p = 0.048). Intriguingly, the relative abundance of periodontal pathogens [*Tannerella forsythia* (p = 0.021), *Treponema denticola* (p < 0.001), and *Campylobacter rectus* (p = 0.039)] was significantly reduced two weeks after PTOR (Fig. 2C). Species-level salivary and plaque microbial profiling sorted by individual participant seen in Additional file 1: Figures S4 and S5.

### Altered oral microbial functional pathways following PTOR

**Fig. 3:**
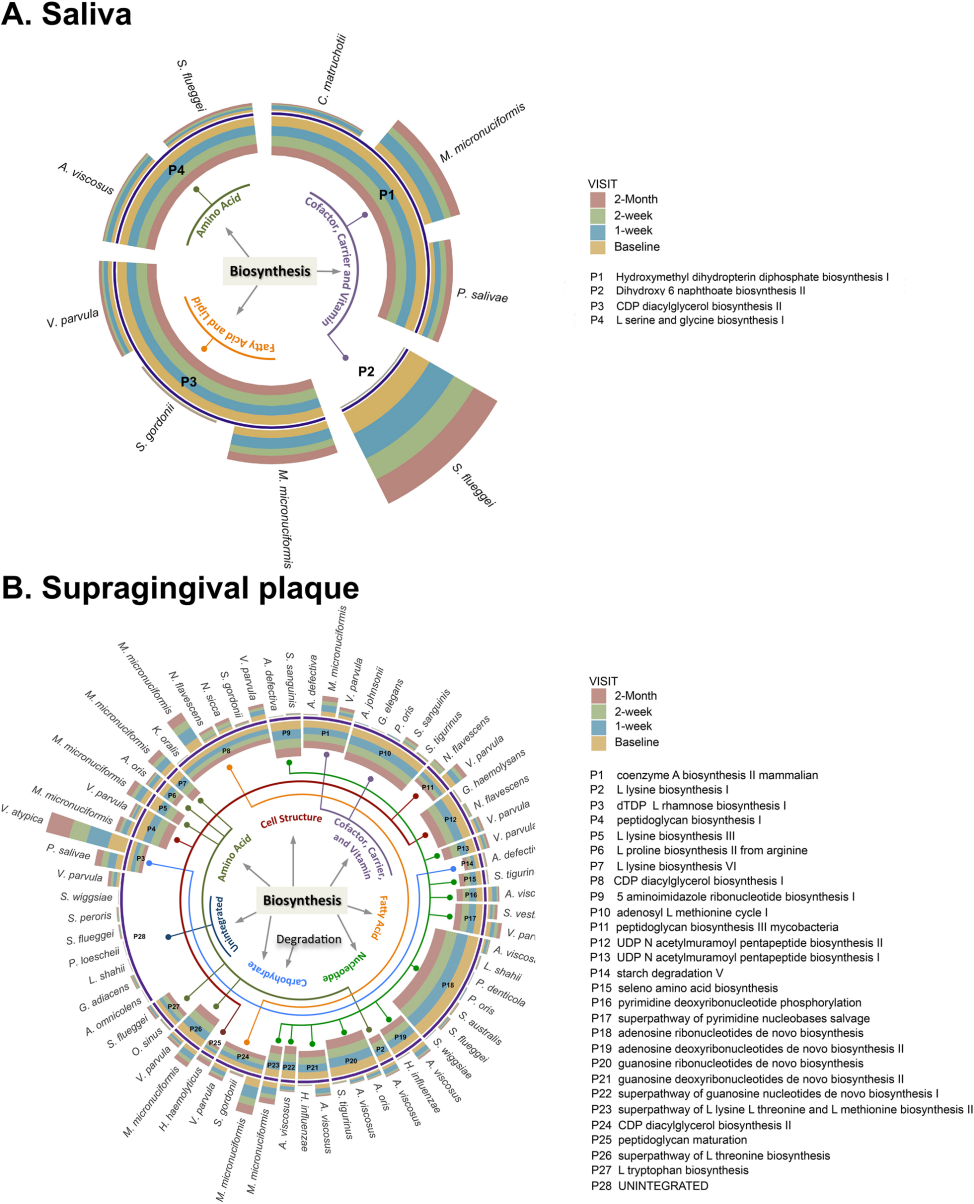
Changes of oral microbial abundance and functional pathway following Prenatal Total Oral Rehabilitation (PTOR). **A** Changes of oral microbial abundance and functional pathway in saliva. **B** Changes of oral microbial abundance and functional pathway in supragingival plaque. To determine differentially abundant (DA) pathways and the corresponding DA taxa, an MMRM with an unstructured covariance matrix was used for each pathway; for an identified DA pathway, an MMRM with an unstructured covariance matrix was used for each taxon to determine DA taxa. All analyses included race, Decayed teeth (DT), frequency of brushing, and BOP as covariates. For multiple testing correction, the Benjamini–Hochberg procedure was used to control false discovery rate (FDR) at a 5% level. Arcs in different colors correspond to different superclasses. For instance, the orange-colored arc represents superclass “fatty acid” that include pathways P8 and P24

The abundance of multiple metabolic pathways of salivary and plaque microbiome was identified to be significantly different between baseline and post-PTOR visits (Fig. 3A saliva and Fig. 3B plaque). In addition, metagenomic sequencing functional pathway analysis indicated changes occurred not only at the microbial level but also at the functional level. For example, significant changes in the *Actinomyces defectiva*-associated carbohydrate degradation pathway and *Streptococcus gordonii*-associated fatty acid biosynthesis pathway were noted in the plaque microbiome.

### Modified host immune response following PTOR

Overall, the salivary load of three immune markers was significantly different after PTOR compared to the baseline. Factors associated with the salivary load of the immune markers after PTOR over time (mixed effect model) was assessed. For significant findings, see Additional file 1: Table S1. Among the panel of 25 salivary immune markers, two immune markers are related to adverse birth outcomes, which showed significant differences after receiving PTOR. Interferon-inducible T-cell alpha chemoattractant (ITAC), which negatively correlates with preeclampsia severity, significantly increased at V2. Monocyte chemoattractant protein-1 (MCP-1), which attracts or enhances the expression of other inflammatory factors and cells, is positively correlated with gestational age and was elevated in saliva following PTOR.

**Fig. 4:**
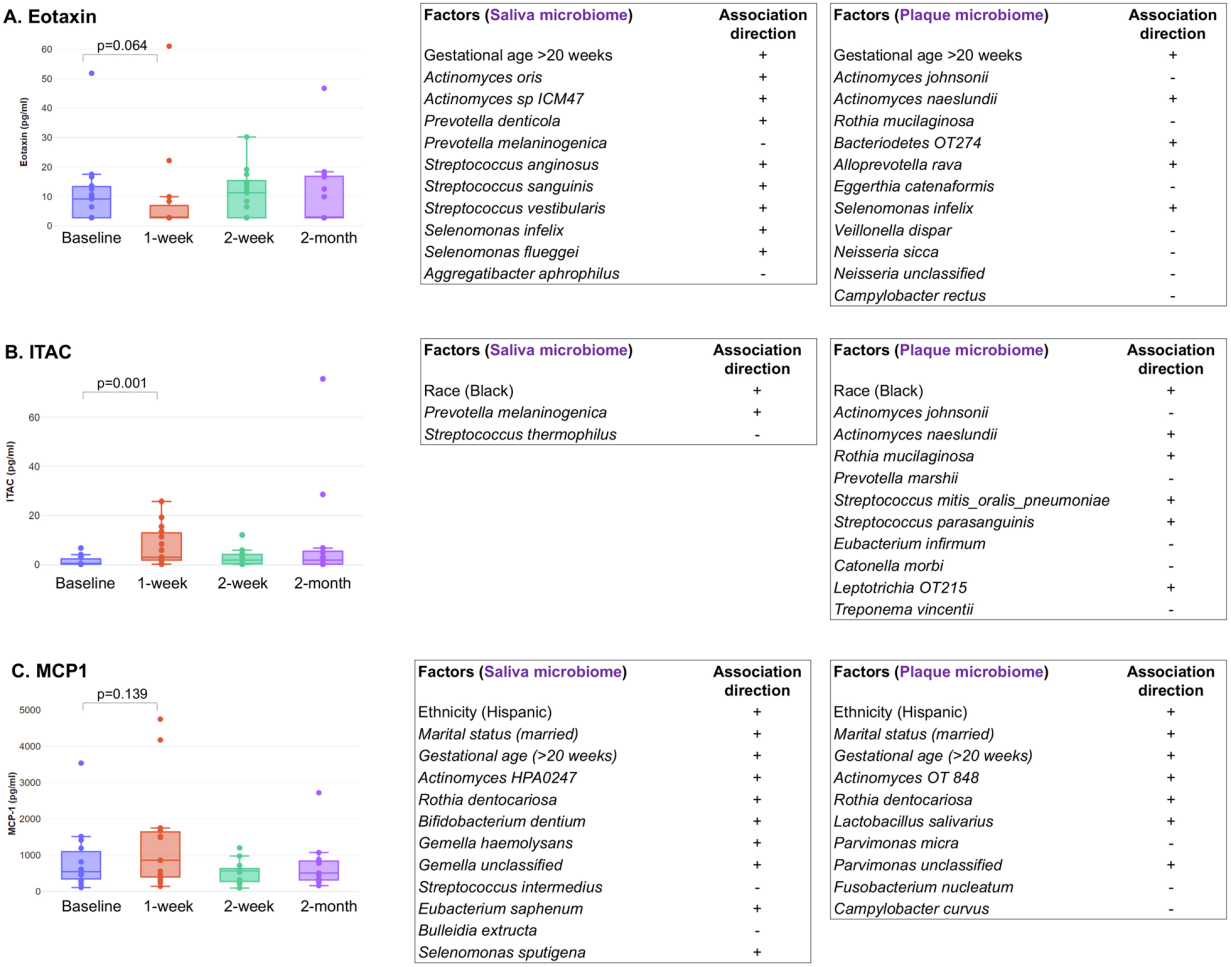
Levels of salivary immune markers following Prenatal Total Oral Rehabilitation (PTOR). **A** Eotaxin levels in saliva **B** ITAC Eotaxin levels in saliva, and **C** MCP1 Eotaxin levels in saliva. To assess the PTOR-related longitudinal association between immune markers and possibly related factors, we first fitted linear mixed effects models with random intercepts for participants of salivary cytokine levels (pg/ml) of immune markers over time on demographic and clinic factors. The model allows us to compare the cytokine levels of immune markers at follow-up visits to those at baseline by setting the time points as categorical. Other variables included were race, education, vaginal yeast infection, work status, whether brushing teeth twice daily, gestational age (> = 20 weeks), ICDAS level, BOP, pain score, and the number of decayed teeth. We then assessed the association between the microbial species and the immune markers using Lasso-penalized linear mixed effects models with random intercepts using saliva and plaque samples, respectively. The results are shown in tables on the right side for each immune marker. The microbiome data in both samples, together with the same set of covariates as in the previous models, were used as candidate variables. The tuning parameters were chosen using Jones’ BIC criterion for longitudinal data

Furthermore, we fitted a Lasso penalized linear fixed effects model for assessing the longitudinal association between each of the ten immune markers and its most relevant demographic/clinic factors and oral microorganisms among ~ 300 candidate variables in saliva and plaque samples. The number of selected variables depends on the tuning parameters that were chosen with Jones’ BIC for longitudinal data (29), which calculates the “effective sample size” based on the covariance matrix of the mixed model. As expected, the “effective sample size” is between independent observations and perfectly linearly correlated observations within each participant. Figure 4 shows that each immune marker was associated with the same demographic or clinical variables but with different salivary and plaque microorganisms. For example, Eotaxin is positively associated with gestational age in both saliva and plaque models but with different microorganisms in those samples.

## Discussion/conclusion

Maternal oral health impacts children’s oral health, including the transmission of oral microorganisms from mothers to children in early life [15]. Therefore, reducing maternal oral pathogens during pregnancy is critical since it could potentially reduce or delay the colonization of oral pathogens in the infant’s oral cavity. Interestingly, although studies from us and others [3, 25, 26] have demonstrated that receiving atraumatic dental restorative treatment during pregnancy led to a significant reduction of *S. mutans* carriage, and receiving periodontal treatment such as scaling and root planning decreased the oral carriage of periodontal pathogen level, the impact of prenatal oral health rehabilitation on the oral microbiota in a whole remains unclear. This pilot study, to our knowledge, is the first study that comprehensively assessed the changes in the oral microbiome and salivary immune responses following PTOR among pregnant women. PTOR was associated with an abundance reduction of periodontal pathogens in supragingival plaque, for instance, a lower relative abundance of *T. forsythia* and *T. denticola* at two weeks compared to the baseline. In addition, the diversity of the plaque microbial community was significantly reduced following PTOR at a 1-week follow-up. Furthermore, metagenomic sequencing functional pathway analysis indicated changes occurred at the microbial and functional levels. We observed significant changes in the *A. defectiva-associated* carbohydrate degradation pathway and *S. gordonii*-associated fatty acid biosynthesis pathway.

Another study highlights the assessment of immune response following PTOR and the association between immune markers and oral microbiome. Since limited previous research has revealed the association between immune response and oral microbiome, we surveyed a panel of 25 immune markers to understand the potential association. These markers have recently been assessed by a cross-sectional study among 5–10 years old children [27], where a significant elevation of salivary cytokines, e.g., IL-6, IL-10, IL-13, IL-15, were found among children with caries. In addition, immune markers, such as IL17 [28], have established roles during anti-fungal immunity. Several salivary immune markers (Eotaxin, MCP-1, and ITAC) were associated with PTOR treatment in this study. Worth noting that the statistical analysis regarding the immune markers was adjusted for the baseline gestational age. We grouped the gestational age into two groups, before and after 20 weeks. The immune markers reported in Fig. 4, Eotaxin, MCP1, and ITAC, were not affected by mothers’ baseline gestational age.

### Eotaxin

Human Eotaxin is a 74 amino acid polypeptide, non-glycosylated protein chemokine which is expressed by a variety of cells, including endothelial cells, epithelial cells, broncho-alveolar macrophages, lung, and dermal fibroblasts, smooth muscle cells, and chondrocytes [29]. Increased levels of circulating eosinophils are pathognomic characteristics for several allergic, inflammatory disorders, and malignancies [30]. Paque and colleagues identified four potential salivary biomarkers (IL-4, 13, 2-ra, and chemokine Eotaxin/CCl11) as discriminatory and could be used to determine the caries susceptibility of an individual. Elevated levels of all four biomarkers for the caries-affected patient were observed, including chemokine Eotaxin (1.7 pg/mL ± 0.9 in patients with deep caries) [31]. Contrarily, the authors pointed out that bacterial composition was not as helpful in classifying caries compared to cytokine/chemokines assessments. These biomarker assays may help clinicians classify the patient and monitor treatment responses by tracking the oral microenvironment health [31]. Queiroz et al. demonstrated that serum levels of inflammatory biomarkers such as Eotaxin could distinguish periodontitis patients from healthy controls. Significantly higher serum levels were detected in patients with chronic periodontitis compared to healthy controls (p = 0.0124) [32]. Our study identified a reduced salivary Eotaxin level at the 1-week follow-up post-PTOR, which might indicate an improved oral health status, including restored caries and improved periodontal conditions from receiving PTOR.

### Monocyte chemoattractant protein 1

MCP-1 is a CC chemokine that was first characterized based on its properties to recruit monocytes into active inflammatory sites [33]. They are expressed either constitutively or induced by cytokines, growth factors, or oxidative stresses [33]. MCP-1 is also overexpressed in metastatic invasion and has an unfavorable prognosis in esophageal squamous cell carcinoma [34] and head and neck squamous cell carcinoma [35]. Although increased serum and gingival crevicular fluid levels of MCP-1 can serve as a diagnostic biomarker for periodontal infection, including aggressive periodontitis [36, 37], Yeates et al. reported that higher concentrations of MCP-1 predicted a longer gestational age, suggest that these cytokines have actions that could prevent early parturition, perhaps through regulatory effects on the chemotaxis of other immune cells [38].

### Interferon-inducible T-cell-alpha chemoattractant (ITAC)

ITAC cytokine (also known as CXCL-11) is expressed by CXR3 receptors. They are secreted by various cells such as monocytes, endothelial cells, and fibroblasts when induced by INF-γ and TNF-α [39]. It is primarily involved in Th1-mediated immune responses and is thereby implicated in autoimmune disorders. Due to their angiogenic functions, they have been implicated in tumor invasion and advancement [40]. Serum levels of ITAC and CXCL13 were positively correlated in patients with primary Sjogren’s syndrome who exhibited continuing disease activity [41]. Owing to its role in T-cell-mediated inflammatory responses, significantly higher levels of ITAC have been detected in tissue samples of patients with oral lichen planus compared with healthy mucosal tissue [42]. Compared to controls, severe preeclamptic women displayed significantly lower ITAC and IP-10 in the umbilical cord plasma samples at the near-term gestation period [43]. Preeclamptic cases displayed significantly lower median plasma umbilical artery and vein levels of ITAC compared to controls, 2.0 vs. 13.9 and 11.9 vs. 31.6 pg/mL, p < 0.05, in artery and vein, respectively. In our study, PTOR is associated with an elevated ITAC at 1-week follow-up, which could signify a potentially reduced risk for preeclampsia.

More interestingly, our results revealed a correlation between oral microorganisms and salivary immune marker levels. The number of different factors was determined to be of importance in predicting the outcome of immune markers before and after PTOR visits using the LASSO-penalized linear mixture models. There was a noticeable difference between the immune marker levels pre- and post-PTOR, though this change is restricted to the week following PTOR, as afterward, markers are largely restored to their baseline levels (Fig. 4). This may indicate that the influence of PTOR in these markers is effective but short-term. A positive sign for a factor indicates that the factor in question (or an increase in the level of the microbiota detected) is associated with an increase in the immune marker, whereas a negative sign is associated with a decrease. For example, being married is associated with an increased presence of the immune marker MCP-1 (Fig. 4C). One finding of note during the analysis was in modeling the immune marker Eotaxin, specifically for the salivary sample. The correlation between the factor gestational age and Prevotella melaninogenica was determined to be negative with a correlation coefficient of -0.43, whereas the correlation between gestational age and Eotaxin was positive at value of 0.30.

### Potential mechanism of PTOR on mothers’ and children’s oral health

**Fig. 5:**
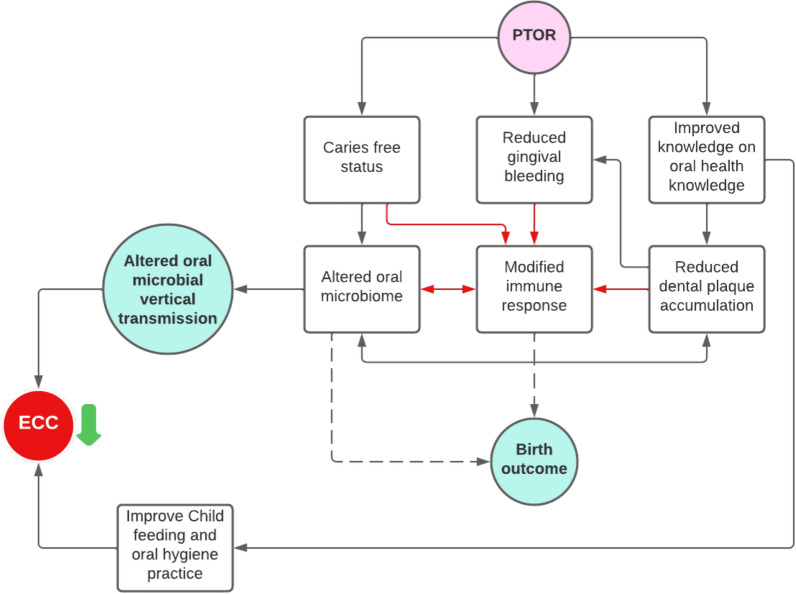
Hypothetic effect of Prenatal Total Oral Rehabilitation (PTOR) on mother’s and child’s oral health. We propose the hypothetic model of PTOR’s effect. First, PTOR helps pregnant women achieve caries-free status before delivery, which is potentially associated with an altered oral microbial community including fewer pathogenic organisms and plausible changes in immune markers. PTOR is also potentially related to a reduced vertical transmission of oral pathogens from mothers to their infants. Moreover, PTOR reduces gingival inflammation, potentially associated with host immune response, and in turn, affects the oral microbiome. Finally, women received PTOR have increased knowledge in perinatal oral health, which would improve their oral hygiene practice, and their children’s feeding, oral hygiene practice, and early infancy dental care utilization. Collectively, providing PTOR could potentially reduce early childhood caries onset

As we continue the follow-up for the children whose mothers received PTOR, we propose the following hypothetic model (Fig. 5) and plan to confirm our findings and test hypotheses based on this model in a large-scale trial. First, PTOR helps pregnant women achieve caries-free status before delivery, which is potentially associated with an altered oral microbial community, including fewer pathogenic organisms and favorable changes in immune markers. PTOR is also potentially related to reduced vertical transmission of oral pathogens from mothers to their infants. Second, PTOR reduces gingival inflammation, potentially associated with the host immune response, and in turn, affects the oral microbiome. Finally, PTOR helps women increase their knowledge in perinatal oral health, which would improve their oral hygiene practice and their children’s feeding, oral hygiene practice, and early infancy dental care utilization. Collectively, providing PTOR has the chance to reduce early childhood caries (ECC) onset. In this pilot study, we only tested the effect of PTOR on mothers’ oral health and microbial changes during pregnancy. Due to the study design with 2-month follow up period, and the small sample size, the study is not powered to detect the birth outcome difference between the PTOR group and the general population. The long-term effect of PTOR on mothers’ oral health, children’s oral health, and birth outcomes deserve further investigation in well-designed clinical trials.

### Limitations

The following limitations need to be considered when interpreting our study results: (1) Limited sample size that hampers further stratification of factors, such as assessment of baseline gestational age on oral microbiome changes following PTOR; (2) The study was only conducted in one US city. Thus, generalization to other populations is unreliable due to the small convenient sample size.

## Conclusions

PTOR, a clinical regimen that targets the critical prenatal period and fully restores women’s oral health to a "disease-free status" before the delivery, is associated with the alteration of the oral microbiome and immune response among a cohort of underserved US pregnant women. Therefore, future randomized clinical trials are warranted to comprehensively assess the impact of PTOR on the maternal oral flora, birth outcomes, and their offspring's oral health.

## Supplementary Information

**Additional file 1: Figure S1.** Library size. **Figure S2.** Median Quality Scores. **Figure S3.** Rarefaction curve. **Figure S4.** Species-level salivary microbial profiling (sorted by individual participant). **Figure S5.** Species-level plaque microbial profiling (sorted by individual participant). **Table S1.** Factors associated with immune marker levels (mixed effect model).

## Data Availability

The sequences have been submitted to the NCBI Bioproject database under accession number PRJNA852341 and SUB11646447. All data generated or analyzed during this study are included in this article. Further enquiries can be directed to the corresponding author.

## References

[1] Xiao J, Alkhers N, Kopycka-Kedzierawski DT, Billings RJ, Wu TT, Castillo DA, Rasubala L, Malmstrom H, Ren Y, Eliav E (2019). Prenatal oral health care and early childhood caries prevention: a systematic review and meta-analysis. Caries Res.

[2] Xiao J, Kopycka-Kedzierawski DT, Billings RJ (2019). National dental practice-based research network collaborative G: intergenerational task: helping expectant mothers obtain better oral health care during pregnancy. J Am Dent Assoc.

[3] Jang H, Al Jallad N, Wu TT, Zeng Y, Fadaak A, Malmstrom H, Fiscella K, Xiao J (2021). Changes in candida Albicans, streptococcus Mutans and oral health conditions following prenatal total oral rehabilitation among underserved pregnant women. Heliyon.

[4] de Araujo Figueiredo SC, Goncalves Carvalho Rosalem C, Costa Cantanhede AL, Abreu Fonseca Thomaz EB, Nogueira Fontoura, da Cruz MC (2017). Systemic alterations and their oral manifestations in pregnant women. J Obstet Gynaecol Res.

[5] Corbella S, Taschieri S, Francetti L, De Siena F, Del Fabbro M (2012). Periodontal disease as a risk factor for adverse pregnancy outcomes: a systematic review and meta-analysis of case-control studies. Odontology.

[6] Puertas A, Magan-Fernandez A, Blanc V, Revelles L, O'Valle F, Pozo E, Leon R, Mesa F (2018). Association of periodontitis with preterm birth and low birth weight: a comprehensive review. J Matern Fetal Neonatal Med.

[7] Chaffee BW, Gansky SA, Weintraub JA, Featherstone JD, Ramos-Gomez FJ (2014). Maternal oral bacterial levels predict early childhood caries development. J Dent Res.

[8] Fisher-Owens SA, Gansky SA, Platt LJ, Weintraub JA, Soobader MJ, Bramlett MD, Newacheck PW (2007). Influences on children’s oral health: a conceptual model. Pediatrics.

[9] Iida H (2017). Oral health interventions during pregnancy. Dent Clin North Am.

[10] Xiao J, Fiscella KA, Gill SR (2020). Oral microbiome: possible harbinger for children’s health. Int J Oral Sci.

[11] Aagaard K, Ma J, Antony KM, Ganu R, Petrosino J, Versalovic J (2014). The placenta harbors a unique microbiome. Sci Transl Med.

[12] Bearfield C, Davenport ES, Sivapathasundaram V, Allaker RP (2002). Possible association between amniotic fluid micro-organism infection and microflora in the mouth. BJOG.

[13] Gomez-Arango LF, Barrett HL, McIntyre HD, Callaway LK, Morrison M, Nitert MD (2017). Contributions of the maternal oral and gut microbiome to placental microbial colonization in overweight and obese pregnant women. Sci Rep.

[14] Douglass JM, Li Y, Tinanoff N (2008). Association of mutans streptococci between caregivers and their children. Pediatr Dent.

[15] Xiao J, Moon Y, Li L, Rustchenko E, Wakabayashi H, Zhao X, Feng C, Gill SR, McLaren S, Malmstrom H (2016). Candida albicans carriage in children with severe early childhood caries (S-ECC) and maternal relatedness. PLoS ONE.

[16] Thompson TA, Cheng D, Strobino D (2013). Dental cleaning before and during pregnancy among Maryland mothers. Matern Child Health J.

[17] Marchi KS, Fisher-Owens SA, Weintraub JA, Yu Z, Braveman PA (2010). Most pregnant women in California do not receive dental care: findings from a population-based study. Public Health Rep.

[18] Singhal A, Chattopadhyay A, Garcia AI, Adams AB, Cheng D (2014). Disparities in unmet dental need and dental care received by pregnant women in Maryland. Matern Child Health J.

[19] Shivakumar K, Prasad S, Chandu G (2009). International caries detection and assessment system: a new paradigm in detection of dental caries. J Conservat Dent JCD.

[20] Loe H (1967). The gingival index, the plaque index and the retention index systems. J Periodontol.

[21] Molenberghs G, Thijs H, Jansen I, Beunckens C, Kenward MG, Mallinckrodt C, Carroll RJ (2004). Analyzing incomplete longitudinal clinical trial data. Biostatistics.

[22] Anderson MJ (2001). A new method for non-parametric multivariate analysis of variance. Austral Ecol.

[23] Benjamini Y, Hochberg Y (1995). Controlling the false discovery rate—a practical and powerful approach to multiple testing. J R Stat Soc B.

[24] Jones RH (2011). Bayesian information criterion for longitudinal and clustered data. Stat Med.

[25] Volpato FC, Jeremias F, Spolidório DM, Silva SR, Valsecki Junior A, Rosell FL (2011). Effects of oral environment stabilization procedures on Streptococcus mutans counts in pregnant women. Braz Dent J.

[26] Asad R, Ali Khan KA, Javed T, Arshad MB, Chaudhary A, Khan AA (2018). Effect of atraumatic restorative treatment on streptococcus mutans count in saliva of pregnant women: a randomized controlled trial. Ann King Edward Med Univ.

[27] Baker JL, Morton JT, Dinis M, Alvarez R, Tran NC, Knight R, Edlund A (2021). Deep metagenomics examines the oral microbiome during dental caries, revealing novel taxa and co-occurrences with host molecules. Genome Res.

[28] Mengesha BG, Conti HR (2017). The Role of IL-17 in protection against mucosal candida infections. J Fungi (Basel).

[29] Rankin SM, Conroy DM, Williams TJ (2000). Eotaxin and eosinophil recruitment: implications for human disease. Mol Med Today.

[30] Zajkowska M, Mroczko B (2021). From allergy to cancer-clinical usefulness of eotaxins. Cancers (Basel).

[31] Paqué PN, Herz C, Wiedemeier DB, Mitsakakis K, Attin T, Bao K, Belibasakis GN, Hays JP, Jenzer JS, Kaman WE (2021). Salivary biomarkers for dental caries detection and personalized monitoring. J Pers Med.

[32] de Queiroz AC, Taba M, O'Connell PA, da Nóbrega PB, Costa PP, Kawata VK, Trevisan GL, Novaes AB, de Souza SL, Palioto DB (2008). Inflammation markers in healthy and periodontitis patients: a preliminary data screening. Braz Dent J.

[33] Deshmane SL, Kremlev S, Amini S, Sawaya BE (2009). Monocyte chemoattractant protein-1 (MCP-1): an overview. J Interferon Cytokine Res.

[34] Koide N, Nishio A, Sato T, Sugiyama A, Miyagawa S (2004). Significance of macrophage chemoattractant protein-1 expression and macrophage infiltration in squamous cell carcinoma of the esophagus. Am J Gastroenterol.

[35] Ji WT, Chen HR, Lin CH, Lee JW, Lee CC (2014). Monocyte chemotactic protein 1 (MCP-1) modulates pro-survival signaling to promote progression of head and neck squamous cell carcinoma. PLoS ONE.

[36] Pradeep AR, Daisy H, Hadge P, Garg G, Thorat M (2009). Correlation of gingival crevicular fluid interleukin-18 and monocyte chemoattractant protein-1 levels in periodontal health and disease. J Periodontol.

[37] Gunpinar S, Alptekin NO, Dundar N (2017). Gingival crevicular fluid levels of monocyte chemoattractant protein-1 in patients with aggressive periodontitis. Oral Dis.

[38] Yeates AJ, McSorley EM, Mulhern MS, Spence T, Crowe W, Grzesik K, Thurston SW, Watson GE, Myers GJ, Davidson PW (2020). Associations between maternal inflammation during pregnancy and infant birth outcomes in the seychelles child development study. J Reprod Immunol.

[39] Fallahi P, Ferrari SM, Ragusa F, Ruffilli I, Elia G, Paparo SR, Antonelli A (2020). Th1 chemokines in autoimmune endocrine disorders. J Clin Endocrinol Metab.

[40] Tokunaga R, Zhang W, Naseem M, Puccini A, Berger MD, Soni S, McSkane M, Baba H, Lenz HJ (2018). CXCL9, CXCL10, CXCL11/CXCR3 axis for immune activation—a target for novel cancer therapy. Cancer Treat Rev.

[41] Nocturne G, Seror R, Fogel O, Belkhir R, Boudaoud S, Saraux A, Larroche C, Le Guern V, Gottenberg JE, Mariette X (2015). CXCL13 and CCL11 serum levels and lymphoma and disease activity in primary Sjögren's syndrome. Arthritis Rheumatol.

[42] Marshall A, Celentano A, Cirillo N, McCullough M, Porter S (2017). Tissue-specific regulation of CXCL9/10/11 chemokines in keratinocytes: implications for oral inflammatory disease. PLoS ONE.

[43] Chedraui P, Solís EJ, Pérez-López FR, Schatz F, Kayisli U, Escobar GS, Loja-Chango R, Hidalgo L, Lockwood CJ (2016). Umbilical cord plasma interferon-induced protein 10 (IP-10) and interferon-induced T-cell alpha chemoattractant (ITAC) levels are lower in women with severe preeclampsia. J Perinat Med.

