# Supplementary material for: Metagenomic analysis examines oral microbiome changes and interplay with immune response following prenatal total oral rehabilitation
Source: J Transl Med. 2023 Mar 4;21:172. doi: 10.1186/s12967-023-03997-9 (PMC9985285; doi:10.1186/s12967-023-03997-9)
Supplement: Supplementary file 1 — Additional file 1: Figure S1. Library size. Figure S2. Median Quality Scores. Figure S3. Rarefaction curve. Figure S4. Species-level salivary microbial profiling (sorted by individual participant). Figure S5. Species-level plaque microbial profiling (sorted by individual participant). Table S1. Factors associated with immune marker levels (mixed effect model). [file 12967_2023_3997_MOESM1_ESM.docx]

**Title: Metagenomic Analysis Examines Oral Microbiome Changes and Interplay with Immune Response Following Prenatal Total Oral Rehabilitation**

**Figure S1. Library size**

**(A) Saliva (B) Plaque**

**
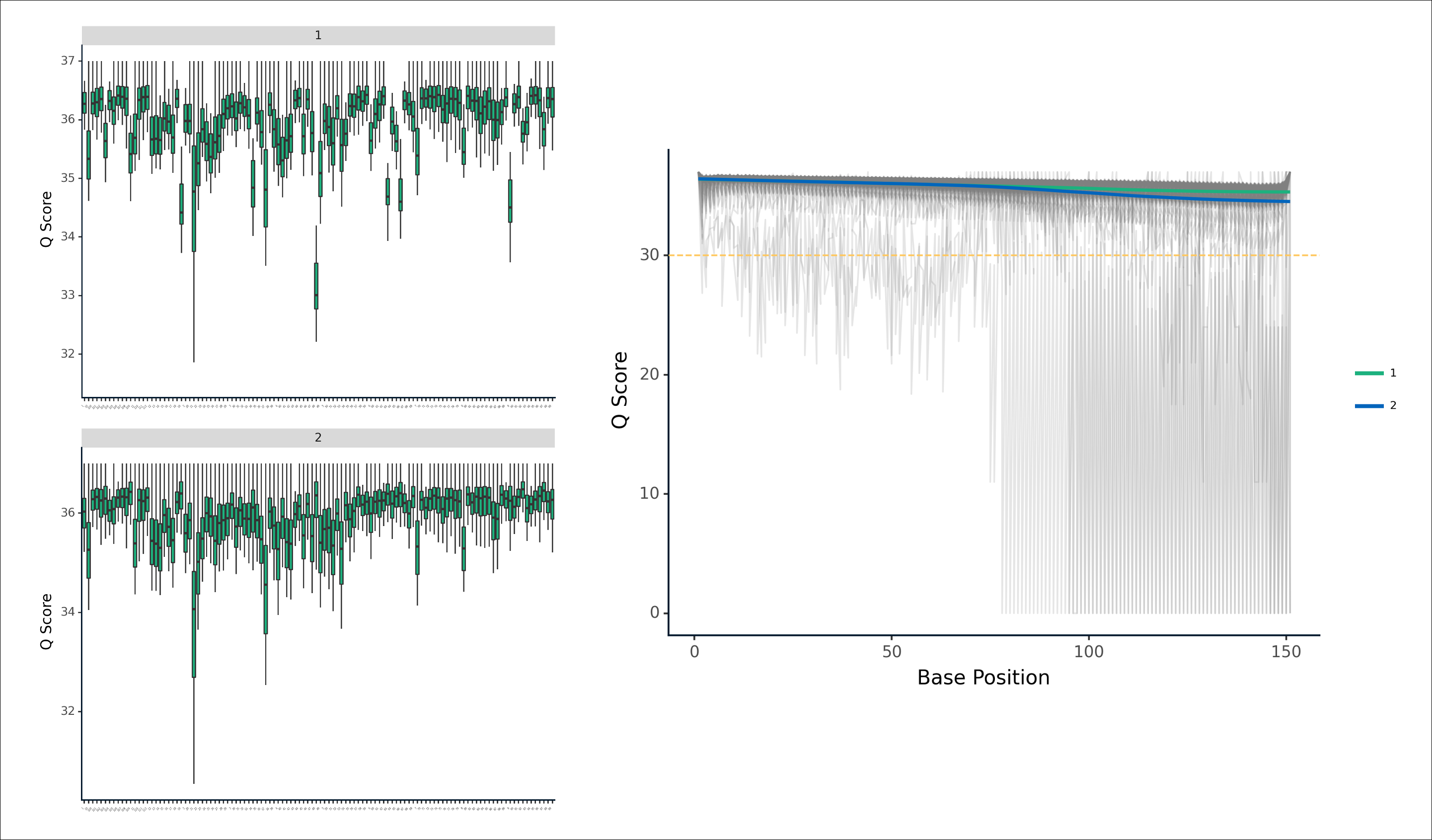
Figure S2. Median Quality Scores.**

Trimmed and quality-filtered fastqs were used to calculate the median quality scores and the sequence quality per base for each sample. quality scores above 25 are generally considered acceptable.


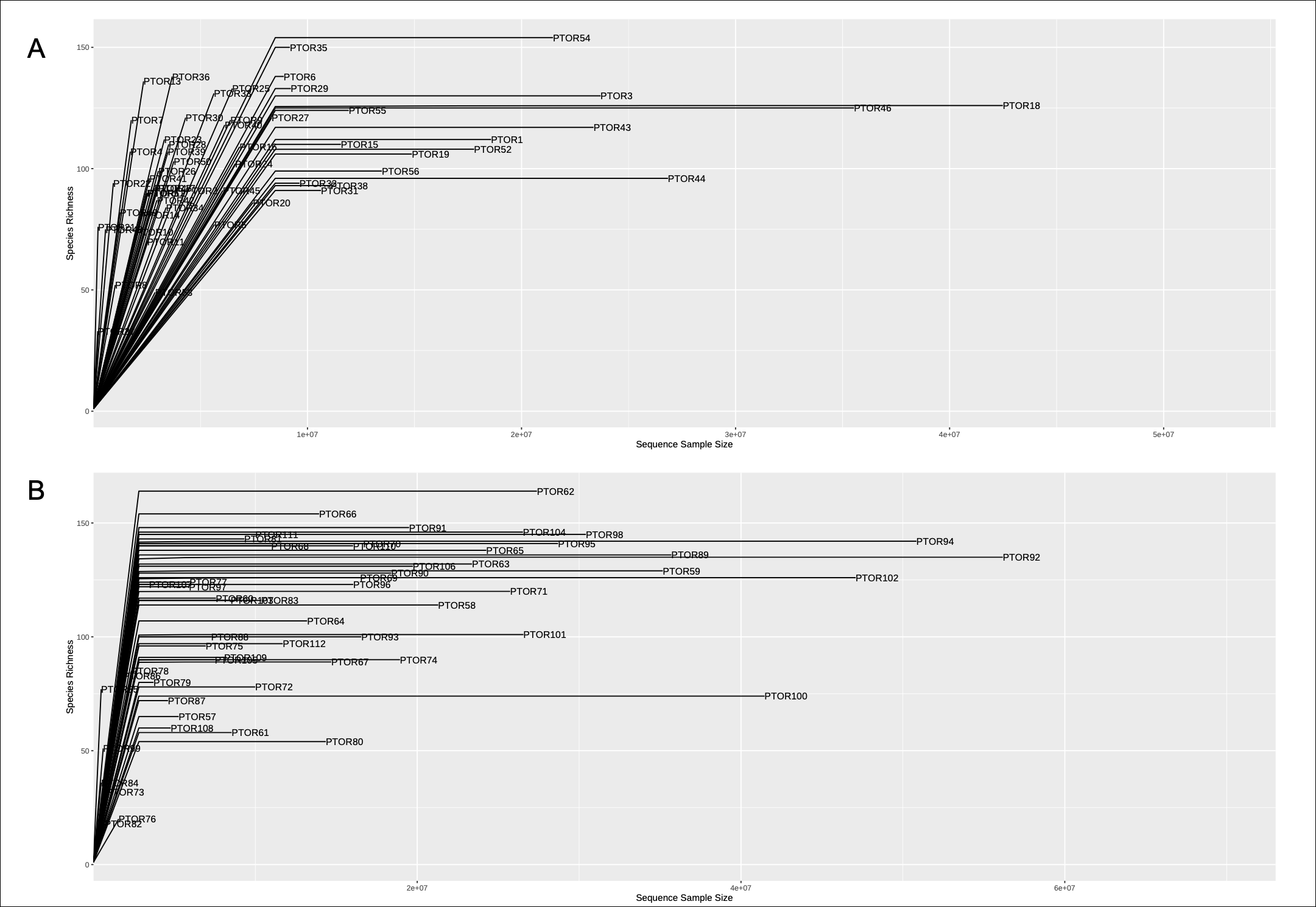
**Figure S3. Rarefaction curve**

**(A) Saliva (B) Plaque**

**Figure S4. Species-level salivary microbial profiling (sorted by individual participant)**

The species shown here are the top 50 most abundant taxonomic groups overall plus the top 10 most abundant for each sample

**Figure S5. Species-level plaque microbial profiling (sorted by individual participant)**

The species shown here are the top 50 most abundant taxonomic groups overall plus the top 10 most abundant for each sample

**Table S1 Factors associated with immune marker levels (mixed effect model)**

| **Immune**  **marker**  **Factors** | **Eotaxin** | | | **MDC*** | | | **IL 15** | | | **IL 1ra** | | | **IL 1a** | | | **IP 10*** | | | **MCP 1** | | | **ITAC*** | | | **IL 1b** | | |
| --- | --- | --- | --- | --- | --- | --- | --- | --- | --- | --- | --- | --- | --- | --- | --- | --- | --- | --- | --- | --- | --- | --- | --- | --- | --- | --- | --- |
|  | Est | SE | P | Est | SE | P | Est | SE | P | E st | SE | P | Est | SE | P | Est | SE | P | Est | SE | P | Est | SE | P | Est | SE | P |
| **Visit 2 vs Visit 1** | -0.54 | 0.28 | **0.06** | -0.19 | 0.16 | 0.24 | -0.26 | 0.21 | 0.21 | -0.03 | 0.06 | 0.56 | 0.11 | 0.16 | 0.48 | 0.87 | 0.31 | **0.01** | 0.36 | 0.23 | 0.14 | 2.08 | 0.50 | **<0.001** | 0.81 | 0.80 | 0.32 |
| **Visit 3 vs Visit 1** | -0.02 | 0.32 | 0.95 | 0.25 | 0.18 | 0.15 | 0.14 | 0.23 | 0.55 | -0.10 | 0.06 | 0.10 | -0.17 | 0.18 | 0.36 | -0.12 | 0.35 | 0.73 | -0.20 | 0.27 | 0.46 | 1.03 | 0.55 | **0.07** | -0.68 | 0.89 | 0.45 |
| **Visit 4 vs Visit 1** | -0.27 | 0.32 | 0.40 | 0.19 | 0.18 | 0.28 | 0.05 | 0.23 | 0.83 | -0.01 | 0.06 | 0.82 | 0.11 | 0.18 | 0.54 | 0.25 | 0.35 | 0.49 | 0.13 | 0.27 | 0.62 | 1.37 | 0.55 | **0.02** | -.25 | 0.90 | 0.78 |
| **Race (Black vs Non-Black)** | 0.39 | 1.21 | 0.75 | 0.98 | 1.00 | 0.34 | 0.84 | 1.23 | 0.50 | -0.54 | 0.23 | **0.02** | -1.98 | 0.81 | **0.02** | -0.51 | 2.85 | 0.86 | -1.66 | 1.75 | 0.35 | 0.52 | 1.57 | 0.74 | -8.55 | 3.03 | **0.01** |
| **College and above vs below** | -0.11 | 0.85 | 0.90 | -0.08 | 0.70 | 0.91 | 0.25 | 0.86 | 0.77 | -0.08 | 0.16 | 0.61 | -0.15 | 0.57 | 0.79 | 1.54 | 1.97 | 0.44 | 1.01 | 1.21 | 0.41 | 1.98 | 1.13 | **0.09** | 1.45 | 2.15 | 0.51 |
| **Vaginal yest infection (Y/N)** | 0.27 | 0.61 | 0.65 | .11 | 0.39 | 0.78 | 0.14 | 0.47 | 0.77 | -0.06 | 0.09 | 0.51 | -0.83 | 0.31 | **0.01** | -0.03 | 1.09 | 0.98 | 0.15 | 0.67 | 0.82 | -0.27 | 0.61 | 0.66 | -1.55 | 1.17 | 0.20 |
| **Employment (Y/N)** | 0.24 | 0.75 | 0.76 | -0.40 | 0.45 | 0.38 | -0.17 | 0.55 | 0.76 | 0.14 | 0.10 | 0.18 | 0.56 | 0.37 | 0.14 | 0.69 | 1.29 | 0.60 | 0.21 | 0.79 | 0.79 | 1.54 | 0.71 | **0.04** | 3.06 | 1.37 | **0.03** |
| **Brushing teeth twice/daily (Y/N)** | -0.41 | 1.05 | 0.69 | -0.40 | 0.85 | 0.64 | -0.14 | 1.05 | 0.90 | 0.19 | 0.20 | 0.34 | 1.11 | 0.69 | 0.12 | 1.18 | 2.41 | 0.63 | 1.74 | 1.48 | 0.25 | 0.87 | 1.36 | 0.52 | 5.38 | 2.60 | **0.05** |
| **Gestational week>= 20w (Y/N)** | 0.75 | 1.19 | 0.53 | 1.09 | 0.92 | 0.25 | 0.75 | 1.14 | 0.51 | -0.36 | 0.21 | **0.10** | -1.09 | 0.75 | 0.15 | -1.13 | 2.63 | 0.67 | -0.92 | 1.62 | 0.57 | -2.65 | 1.47 | **0.08** | -8.01 | 2.82 | **0.01** |
| **HTN (Y/N)** |  |  |  | 0.11 | 0.65 | 0.86 | 0.13 | 0.80 | 0.87 | 0.08 | 0.15 | 0.59 | 0.11 | 0.53 | 0.83 | 0.57 | 1.84 | 0.76 | 0.59 | 1.13 | 0.61 | -0.36 | 1.06 | 0.73 | 1.63 | 2.02 | 0.43 |
| **ICDAS >3 vs <=3** | 1.16 | 1.30 | 0.38 | 0.87 | 1.00 | 0.39 | 0.83 | 1.24 | 0.51 | -0.42 | 0.23 | **0.08** | -1.85 | 0.81 | **0.03** | -0.61 | 2.85 | 0.83 | -2.27 | 1.75 | 0.20 | -1.74 | 1.59 | 0.28 | -7.87 | 3.06 | **0.01** |
| **Bleeding on probing sites >7 vs <=7** | -0.05 | 0.39 | 0.89 | -0.06 | 0.22 | 0.80 | 0.01 | 0.29 | 0.98 | 0.04 | 0.07 | 0.57 | 0.07 | 0.22 | 0.74 | -0.24 | 0.44 | 0.59 | -0.15 | 0.33 | 0.66 | 0.38 | 0.65 | 0.56 | 1.02 | 1.07 | 0.34 |
| **Pain score** | -0.11 | 0.10 | 0.28 | 0.07 | 0.05 | 0.23 | -0.02 | 0.07 | 0.80 | -0.001 | 0.02 | 0.95 | -0.01 | 0.06 | 0.84 | 0.17 | 0.11 | 0.12 | 0.11 | 0.08 | 0.19 | 0.03 | 0.17 | 0.88 | -0.56 | 0.27 | **0.04** |
| **Decayed teeth** | -0.27 | 0.53 | 0.62 | -0.12 | 0.40 | 0.76 | -0.01 | 0.49 | 0.99 | 0.03 | 0.09 | 0.80 | 0.41 | 0.32 | 0.21 | 0.07 | 1.12 | 0.95 | 0.61 | 0.69 | 0.38 | 0.54 | 0.63 | 0.40 | 1.34 | 1.21 | 0.28 |

**EST estimate**

*** P<0.05**
